# Impact of COVID-19 on the surgical volume of general surgery residents as main surgeons in a National Training Program in Costa Rica: A cross-sectional study

**DOI:** 10.1097/MD.0000000000027041

**Published:** 2021-08-27

**Authors:** Jose Pablo Rivera-Chavarría, Carlos Gutierrez-Lopez, Jose Antonio Castro-Cordero, Gustavo Jimenez-Ramirez

**Affiliations:** a Health Services Administration. Colorectal Surgeon, Mexico Hospital, Caja Costarricense Seguro Social. Professor of General Surgery, University of Costa Rica, Department of General Surgery, Hospital México, La Uruca, San José, Costa Rica; b Mexico Hospital, Caja Costarricense Seguro Social, San José, Costa Rica; c Epidemiology Unit, Mexico Hospital, Caja Costarricense Seguro Social, San José, Costa Rica; d Department of General Surgery, Mexico Hospital, Caja Costarricense Seguro Social, University of Costa Rica, San José, Costa Rica.

**Keywords:** coronavirus, Costa Rica, coronavirus disease 19 Pandemic, general surgery, surgical education, surgical residency, surgical training, virtual education

## Abstract

To quantify the impact of coronavirus disease 19 (COVID-19) on the surgical volume of residents’ medical practice in Costa Rica's General Surgery Residency Program.

The COVID-19 pandemic has caused a significant disruption in people's lives. Health systems worldwide have been forced to adapt to the new normal, which has posed a challenge for medical residency programs, especially in the surgical field.

This transversal study includes the surgical records of all residents of the General Surgery program who worked as main surgeons at the Mexico Hospital of the Costa Rican Social Security between December 23, 2019, and June 25, 2020.

As main surgeons, a total of 10 residents performed 291 pre-pandemic surgeries and 241 pandemic surgeries.

When comparing the distribution of procedures performed by residency levels, it is observed that the postgraduate year -2 increased the number of procedures performed during the pandemic period (pre-pandemic 19% vs pandemic 27%, *P* = .028). There was no statistically significant difference between the pre-pandemic and pandemic periods in the remaining levels.

When comparing the procedures by unit, a statistically significant decrease was observed in the Endocrine-Abdominal Wall Unit (pre-pandemic 18.3% vs pandemic 5.4%, *P* < .001). Conversely, a statistically significant increase was identified in Surgical Emergencies Unit procedures (40.0% vs post 51.7%, *P* = .007). No statistically significant differences were observed in the remaining the Units.

The COVID-19 pandemic had no statistically significant effect on surgeries performed by residents of the General Surgery Residency Program as main surgeons in a national training center in Costa Rica. The Department's timely measures and pro-resident attitude were the key reasons for the above results.

## 1 Introduction

The initial cases of Severe Acute Respiratory Syndrome Coronavirus 2 occurred in Wuhan, Hubei Province, China, between December 2019 and January 2020.^[1]^ By January 20, 2020, the first confirmed case was documented within the United States.^[2]^ In Costa Rica, the first case was confirmed on March 6, 2020.^[3]^ The Coronavirus Disease 19 (COVID-19) pandemic has generated rapid and significant changes in patient care worldwide, especially in the surgical area.^[4]^ The American College of Surgeons recommended the cancellation of elective surgical cases in the United States of America.^[5,6]^ Hospitals, following global tendency, have focused on mitigation strategies, including postponing or canceling elective surgical procedures using triage guidelines, converting clinics to telemedicine visits, and suspending face-to-face meetings, including didactic sessions for trainees.^[4]^

Medical residency programs have also been affected, forcing them to adapt and modify their usual functioning, with surgical programs being the most affected.^[7–13]^ A series of strategies have been implemented to reduce residents’ exposure to COVID-19 at the expense of a reduction in surgical procedures performed by residents.^[4–6,14,15]^

Surgical residencies in Costa Rica are special public programs hosted by the University of Costa Rica and the Costa Rican Social Security, in which residents are both workers and students. The hands-on method is the primary teaching technique, which provides the resident with a significant amount of supervised surgical procedures, improving their skills and experience. In this study, we attempt to quantify the impact of the COVID-19 pandemic on the surgical volume of residents’ medical practice in a National General Surgery Residency Program in Costa Rica.

## 2 Methods

México Hospital is one of Cost Rica's three national reference hospitals, offering a wide range of surgical specialties. Moreover, it is one of the most indispensable resident training centers in the country.

**Figure 1:**
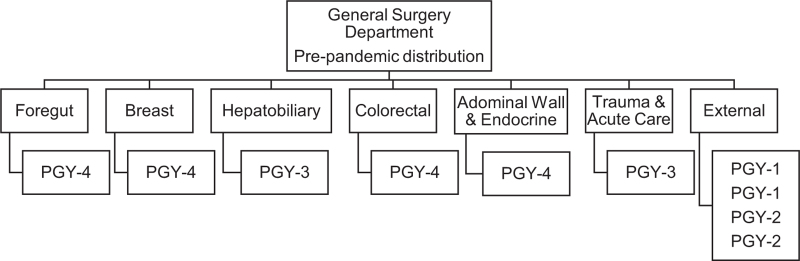
General surgery department pre-pandemic distribution.

The Department of General Surgery of México Hospital is divided into six units: Colorectal Unit; Hepatobiliary Unit; Foregut Unit; Breast Unit; Endocrine-Abdominal Wall Unit; and Surgical Emergencies Unit. We have ten General Surgery residents, two Postgraduate first-year residents (PGY-1), two Postgraduate second-year residents (PGY-2), two Postgraduate third-year residents (PGY-3), and four Postgraduate fourth-year residents (PGY-4). Prior to the pandemic, four residents conducted rotations in other Departments, including Vascular Surgery, Urology, Cardiothoracic Surgery, and Intensive Care. Nonetheless, at the onset of the pandemic, the decision was made to suspend rotations and return all residents to the Department of General Surgery (Fig. 1).

**Figure 2:**
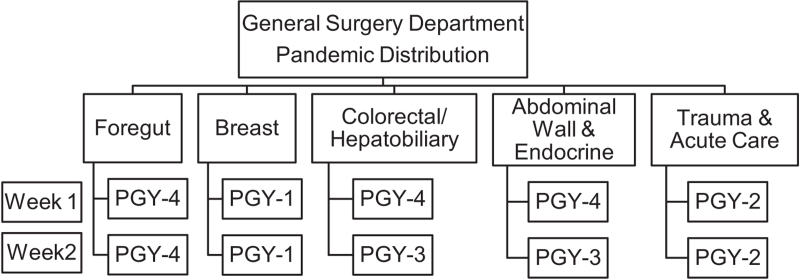
General surgical department pandemic distribution.

The General Surgery Department decided to divide residents into two groups on March 23, 2020, with an equitable distribution of all levels. This measure was implemented to reduce residents’ exposure to Covid-19 and avoid the need for all residents to be isolated in the eventual stages of an infection. Each group covered the six units and essential surgery wards with an average of 3 shifts per week (Fig. 2). As per American College of Surgeons recommendations, all elective non-urgent and non-oncological surgery were suspended.

Additionally, the face-to-face master classes, which were held every Monday at 7:00 hours were replaced with online classes from Monday to Friday at 14:00 hours. Residents who were unable to connect in real-time were given recordings.

Three types of procedures were defined were defined for this study: Oncological, including all malignant pathology from all units; Non-oncological, including benign pathology; and Surgical Emergencies.

The impact of these measures was analyzed by comparing surgical records based on the type of surgery and the resident's level to determine how alterations affected their surgical record as main surgeons, positively or negatively. For the purposes of this study, the pre-pandemic period was defined as that between December 23, 2019, and March 23, 2020, and the pandemic period was defined as that between March 24 and June 25, 2020.

We used descriptive statistics with absolute and relative frequency distributions for the statistical analysis. The Mid-*P* Exact test was used to compare the groups. The chosen level of significance was 0.05. The computer programs used were SPSS version 23 and Past version 4.02.

No ethics committee or institutional review board approval was required, as this research did not include studies on human subjects, human data or tissue, or animals.

## 3 Results

As main surgeons, a total of 10 residents performed 291 pre-pandemic surgeries and 241 pandemic surgeries.

When comparing the distribution of procedures performed by resident level, it was discovered that the PGY-2 increased the number of procedures during the pandemic period (pre-pandemic 19% vs pandemic 27%, *P* = .028). In the remaining levels, no statistically significant difference between the pre and pandemic periods was observed.

When analyzing the procedures performed by units, a decrease in the Endocrine-Abdominal Wall Unit (pre-pandemic 18.3% vs pandemic 5.4%, *P* < .001), an increase in the Surgical Emergencies Unit (40.0% vs post 51.7%, *P* = .007), and no statistically significant differences in the remaining Units were found.

When comparing the procedures by unit and resident level, in PGY-1 and PGY-3, there was no statistically significant difference in any unit. Additionally, in the case of the PGY-3, during the pandemic phase, they performed procedures that they did not execute in the pre-pandemic period (Breast and Foregut Unit). In PGY-4, statistically significant increases were observed in procedures in the Breast Unit (pre-pandemic 1.3% vs. pandemic 8.2%, *P* = .008) and Surgical Emergencies Unit (pre-pandemic 27.1% vs. pandemic 50.9%, *P* < .001), whereas decreases were reported in the Colorectal Unit (pre-pandemic 19.4% vs pandemic 9.1%, *P* = .021) and Endocrine and Abdominal Wall Unit (pre-pandemic 20.6% vs pandemic 4.5%, *P* < .001).

When evaluating the procedures by type, an increase was observed in Surgical Emergencies procedures (pre-pandemic 40.0% vs 53.3% pandemic, *P* = .002) and Oncological surgeries (pre-pandemic 14.8% vs post 21.9%, *P* = .035), and a decrease was observed in Non-oncological surgeries (pre 45.2% vs. post 24.8%, *P* < .001). When comparing by the residency level, no statistically significant changes were observed in PGY-1, PGY-2, and PGY-4; conversely, in PGY-3, an increase was observed in Oncological surgeries (pre 4.0% vs post 29.3%, *P* = .001) (Table 1).

**Table 1:** Surgical procedures according to resident level.

	General					PGY-1					PGY-2					PGY-3					PGY-4				
	Pre-pandemic		Pandemic		*P* value	Pre-pandemic		Pandemic		*P* value	Pre-pandemic		Pandemic		*P* value	Pre-pandemic		Pandemic		*P* value	Pre-pandemic		Pandemic		*P* value
	n	%	n	%		n	%	n	%		n	%	n	%		n	%	n	%		n	%	n	%	
Type of procedure																									
Surgical Emergencies	116	40%	129	53%	.002	20	63%	19	73%	.413	29	54%	37	58%	.843	29	54%	37	58%	.843	42	27%	58	53%	< .001
Non- oncologic	131	45%	60	25%	< .001	10	31%	5	19%	.413	11	20%	18	28%	.660	11	20%	18	28%	.660	88	57%	22	20%	< .001
Oncologic	43	15%	53	22%	.035	2	6%	2	8%	.320	14	26%	9	14%	.341	14	26%	9	14%	.341	25	16%	30	27%	.030
Surgical Unit																									
Breast	17	5,9%	20	8,3%	.285	1	3,1%	1	3,8%	.897	14	25,9%	4	6,3%	.004	0	0,0%	6	14,6%	-	2	1,3%	9	8,2%	.008
Colorectal	34	11,7%	17	7,0%	.068	1	3,1%	1	3,8%	.897	0	0,0%	2	3,1%	.292	3	6,0%	4	9,8%	.533	30	19,4%	10	9,1%	.021
Endocrine-Adbominal wall	53	18,3%	13	5,4%	< .001	7	21,9%	0	0,0%	-	5	9,3%	6	9,4%	.989	9	18,0%	2	4,9%	.063	32	20,6%	5	4,5%	< .001
Surgical Emergencies	116	40,0%	125	51,7%	.007	20	62,5%	19	73,1%	.413	29	53,7%	35	54,7%	.916	26	52,0%	14	34,1%	.094	42	27,1%	56	50,9%	< .001
Foregut	9	3,1%	14	5,8%	.139	0	0,0%	0	0,0%	-	0	0,0%	2	3,1%	-	0	0,0%	3	7,3%	-	9	5,8%	9	8,2%	.460
Hepatobiliary	55	19,0%	46	19,0%	.988	3	9,4%	4	15,4%	.517	5	9,3%	11	17,2%	.225	11	22,0%	12	29,3%	.440	36	23,2%	19	17,3%	.244
Other	6	2,1%	7	2,9%	.554	0	0,0%	1	3,8%	-	1	1,9%	4	6,3%	.283	1	2,0%	0	0,0%	-	4	2,6%	2	1,8%	.720

PGY-1: Postgraduate Year 1. PGY-2: Postgraduate Year 2. PGY-3: Postgraduate Year 3. PGY-4: Postgraduate Year 4.

## 4 Discussion

To the best of our knowledge, this is the first study to examine the number of procedures performed by residents as main surgeons in any specialty. The analysis benefited from the inclusion of all General Surgery residents from the National Program.

The redistribution of residents resulted in two prime benefits. First, it enabled a significant increase in the classes, increasing from one per week to five per week. Second, the introduction of virtual classes allowed a decrease in the residents’ exposure to SARS COV-2 infection. Consequently, only two residents have been infected, and the Units’ performance has not been hindered as a result of prompt isolation.

The reduction observed in the absolute number of surgeries performed by residents during the pandemic period may be justified by canceling all elective procedures. Nevertheless, no statistically significant difference was reported. In general terms, a redistribution of the surgical volume was noted, such as an increase in Oncological and Surgical Emergency procedures at the expense of reduced Non-oncological procedures.

Furthermore, because our Hospital is a training center, and the number of available procedures was limited during the study period, priority was given to residents to perform procedures as main surgeons when possible. As previously mentioned, in the case of the PGY-3, procedures that were not performed by residents in the pre-pandemic period, such as Foregut and Breast surgeries, were assigned to them. This situation denotes that although our center was affected by the pandemic, due to a pro-resident attitude, the impact was considerably reduced. In addition, the only unit that had a decreased surgical volume during the pandemic was the Endocrine and Abdominal Wall Unit, which can be explained by the fact that the majority of its volume comes from elective procedures such as hernias and goiters.

### 4.1 Limitations

The primary limitation of this study is the limited number of residents who participated in the analysis. Compared to other international centers, the number of students seems low. However, the study must be viewed in the context of a representative sample of a National Residency Program in a middle-income country such as Costa Rica. The studied period was between December 2019 and June 2020, as three of the four fourth-year residents completed their residencies on June 31, 2020. Consequently, the analysis was limited to three months, both pre-pandemic and during the pandemic period.

## 5 Conclusions

The Surgical Department's pro-resident attitude and timely measures resulted in a non-statistically significant difference in the number of surgeries performed as main surgeons by General Surgery Residents during the pandemic period.

## Author contributions

**Conceptualization:** Jose Pablo Rivera-Chavarría, Carlos Gutierrez-Lopez, Jose Antonio Castro-Cordero.

**Data curation:** Jose Pablo Rivera-Chavarría, Carlos Gutierrez-Lopez, Jose Antonio Castro-Cordero.

**Formal analysis:** Jose Pablo Rivera-Chavarría, Carlos Gutierrez-Lopez, Jose Antonio Castro-Cordero.

**Investigation:** Jose Pablo Rivera-Chavarría, Carlos Gutierrez-Lopez.

**Methodology:** Jose Pablo Rivera-Chavarría, Jose antonio Castro-Cordero.

**Project administration:** Jose Pablo Rivera-Chavarría.

**Supervision:** Jose Pablo Rivera-Chavarría, Gustavo Jimenez-Ramirez.

**Validation:** Jose Pablo Rivera-Chavarría, Jose Antonio Castro-Cordero, Gustavo Jimenez-Ramirez.

**Visualization:** Jose Pablo Rivera-Chavarría.

**Writing – original draft:** Jose Pablo Rivera-Chavarría, Carlos Gutierrez-Lopez, Jose Antonio Castro-Cordero.

**Writing – review & editing:** Jose Pablo Rivera-Chavarría.
